# Uma análise de custo-efetividade de propofol *versus* midazolam para sedação de pacientes adultos admitidos à unidade de terapia intensiva

**DOI:** 10.5935/0103-507X.20210068

**Published:** 2021

**Authors:** Teresa Raquel Andrade, Jorge Ibrain Figueira Salluh, Raphaela Garcia, Daniela Farah, Paulo Sérgio Lucas da Silva, Danielle F. Bastos, Marcelo Cunio Machado Fonseca

**Affiliations:** 1 AxiaBio Life Sciences International Ltda. - São Paulo (SP), Brasil.; 2 Instituto D’Or de Pesquisa e Ensino - Rio de Janeiro (RJ), Brasil.; 3 Unidade de Terapia Intensiva Pediátrica, Departamento de Pediatria, Hospital do Servidor Público Municipal - São Paulo (SP), Brasil.; 4 Aspen Pharma - São Paulo (SP), Brasil.; 5 Departamento de Ginecologia, Núcleo de Avaliação de Tecnologias em Saúde, Universidade Federal de São Paulo - São Paulo (SP), Brasil.

**Keywords:** Custo-efetividades, Midazolam, Propofol, Adulto, Estado terminal, Respiração artificial, Unidades de terapia intensiva

## Abstract

**Objetivo:**

Construir um modelo de custo-efetividade para comparar o uso de propofol com o de midazolam em pacientes críticos adultos sob uso de ventilação mecânica.

**Métodos:**

Foi construído um modelo de árvore decisória para pacientes críticos submetidos à ventilação mecânica, o qual foi analisado sob a perspectiva do sistema privado de saúde no Brasil. O horizonte temporal foi o da internação na unidade de terapia intensiva. Os desfechos foram custo-efetividade por hora de permanência na unidade de terapia intensiva evitada e custo-efetividade por hora de ventilação mecânica evitada. Foram obtidos os dados do modelo a partir de metanálise prévia. Assumiu-se que o custo da medicação estava incluído nos custos da unidade de terapia intensiva. Conduziram-se análises univariada e de sensibilidade probabilística.

**Resultados:**

Pacientes mecanicamente ventilados em uso de propofol tiveram diminuição de sua permanência na unidade de terapia intensiva e na duração da ventilação mecânica, respectivamente, em 47,97 horas e 21,65 horas. Com o uso de propofol, ocorreu redução média do custo de U$2.998,971 em comparação ao uso do midazolam. A custo-efetividade por hora de permanência na unidade de terapia intensiva evitada e por hora de ventilação mecânica evitada foi dominante, respectivamente, em 94,40% e 80,8% do tempo.

**Conclusão:**

Ocorreu diminuição significante do custo associado ao uso de propofol, no que se refere à permanência na unidade de terapia intensiva e à duração da ventilação mecânica para pacientes críticos adultos.

## INTRODUCTION

Sedatives are frequently employed to improve mechanical ventilation comfort and synchrony in critically ill patients.^(1)^ The current Clinical Practice Guidelines for the Prevention and Management of Pain, Agitation/Sedation, Delirium, Immobility, and Sleep Disruption in Adult Patients in the ICU (PADIS) recommend a sedation strategy that advise against the use of benzodiazepines.^(2)^ However, benzodiazepines are still commonly used, and in fact, the most widely used sedative agents in critically ill adults are propofol and midazolam.^(3, 4)^

Although midazolam is widely used, one of the main midazolam characteristics is its lipophilic character, and this feature influences its metabolism, thus leading to its accumulation in adipose tissues. In addition, midazolam is broken down into active metabolites, which can be stored in the kidney. Such a cumulative effect may play a role in prolonged weaning from mechanical ventilation, as patients present a long time to awakening. Another concern is the increased risk of *delirium* in patients sedated with midazolam and its long-term consequences, such as postintensive care syndrome.^(1, 5)^

On the other hand, propofol, also a widely used sedative in the intensive care unit (ICU), presents rapid onset of action in seconds, with a fast redistribution of the drug to peripheral tissues. These properties allow a patient to quickly recover consciousness after the discontinuation of propofol, even when it is administered for prolonged periods. Hence, propofol in mechanically ventilated patients is associated with a shorter time needed to recover spontaneous breathing.^(5)^ Nonetheless, there is a perception that propofol may have a higher cost than benzodiazepines.^(6)^

Despite the clinical benefits of avoiding benzodiazepine use in mechanically ventilated patients,^(2)^ the economic impact of this choice has not been thoroughly evaluated. Therefore, we aimed to conduct an economic analysis to compare the use of propofol with the use of midazolam in critically ill adult patients under mechanical ventilation admitted to the ICU for over 24 hours.

## METHODS

### Model structure and population

**Figure 1:**
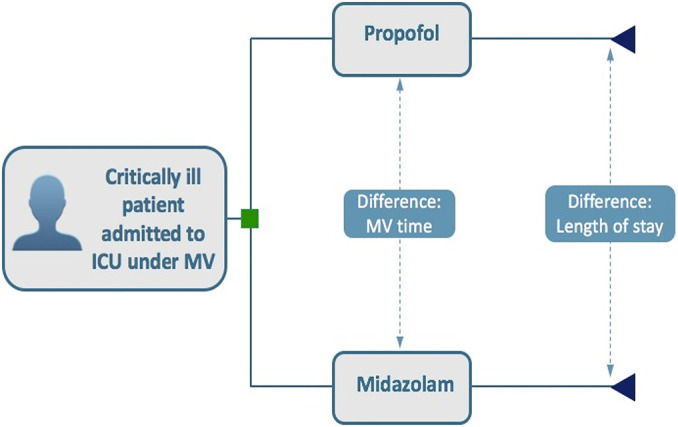
Decision analytical tree model. ICU - intensive care unit; MV - mechanical ventilation.

We developed a decision-tree model to simulate propofol or midazolam administration in critically ill adult patients (≥ 18 years) on mechanical ventilation whose ICU stay exceeded 24 hours (Figure 1).

### Analysis perspective

The perspective of this analysis was the Brazilian supplementary health system (private health system) for 2018.

### Interventions in comparison

The evaluated interventions were two sedatives used in mechanically ventilated patients admitted to the ICU. Propofol, a nonbenzodiazepine drug, and midazolam, a benzodiazepine drug, were compared. In the table 1S (Supplementary material), we present the analgesia management.

### Time horizon

The time horizon corresponds to the period of hospitalization in the ICU of the studies incorporated in the meta-analysis previously carried out by this group. In the studies included in this meta-analysis, the hospitalization period ranged from 224 to 660 hours.^(7)^

Since the time horizon was less than one year, we did not apply a discount rate.

### Clinical data and costs

The clinical data inputs were from a previously published meta-analysis,^(7)^ where the use of propofol reduced ICU stays by 47.97 hours and mechanical ventilation by 21.65 hours.

We used the meta-analysis mean difference of ICU stay and the mean difference of mechanical ventilation days to build the model. Thus, we did not have the number of hours a patient was on mechanical ventilation or the number of hours a patient spent in the ICU for the propofol or midazolam group. Only the time difference between propofol and midazolam use was available for each of these outcomes.

The mean cost of one day in the ICU for a mechanically ventilated adult patient, regardless of the ICU of hospitalization, was retrieved from an insurance database in the state of São Paulo, Brazil.^(8)^

We expressed values as US dollars (US$). The exchange rate in 2018 to convert Brazilian reais (R$) into US dollars was US$1.00 equaled R$3.6552. The mean total cost of one day in an ICU for a mechanically ventilated adult patient was US$ 1,500.42.^(8)^

To calculate the costs, the difference in ICU stay hours implied a cost difference between the arms.

The outcomes of interest evaluated in this model were cost-effectiveness per hour of ICU stay avoided and cost-effectiveness per hour of mechanical ventilation avoided.

### Model assumptions

Our model assumed that the costs of the studied sedatives are included in the patients’ total hospitalization cost. The private health plan database costs represented the private market health costs in Brazil, and the cost of ICU stay per day was the same in both arms.

### Sensitivity analysis

We performed a univariate sensitivity analysis modifying one parameter of the model at a time. Additionally, we carried out a probabilistic sensitivity analysis through a Monte Carlo simulation of ten thousand interactions. In the probabilistic sensitivity analysis, we varied several parameters at the same time. The varied parameters with their respective ranges and references are shown in table 1. We used Palisade @RISK software to execute the sensitivity analyses.

## RESULTS

### Base case

The use of propofol in critically ill patients requiring sedation by mechanical ventilation resulted in a mean reduction of 47.97 hours in the length of ICU stay and 21.65 hours in mechanical ventilation time and a mean decrease of US$ 2,998.97 in the cost when compared to midazolam. Hence, the mean incremental cost-effectiveness ratio (ICER) per hour of ICU stay avoided was US$62.52, and the ICER per hour of mechanical ventilation avoided was US$138.52. Note that the ICER was positive because both the cost and effectiveness differences were negative.

### Sensitivity analysis

In the univariate sensitivity analysis, the parameter that most influenced the cost-effectiveness per hour of ICU stay avoided was the daily cost. In contrast, the parameter that most influenced the cost-effectiveness per hour of mechanical ventilation avoided was ICU length of stay.

**Figure 2:**
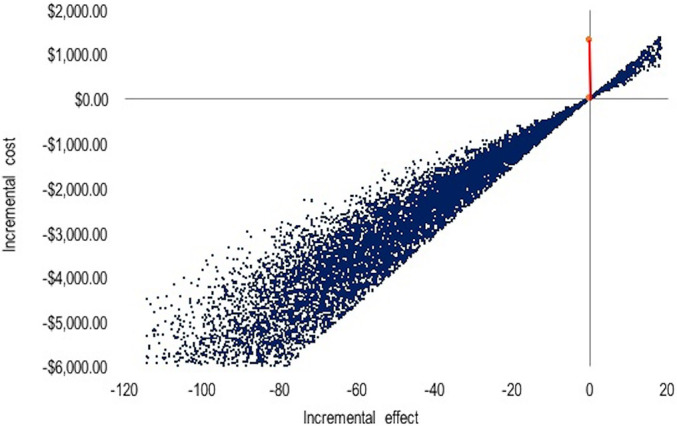
Probabilistic sensitivity analysis for cost-effectiveness per hour of avoided intensive care unit stay with 10,000 interactions.

The probabilistic sensitivity analysis for the cost-effectiveness per hour of ICU stay avoided showed that most of the points (94.4%) were located in the third quadrant of the graph, indicating lower costs and decreased length of ICU stay when patients used propofol. Propofol was the dominant alternative (Figure 2).

**Figure 3:**
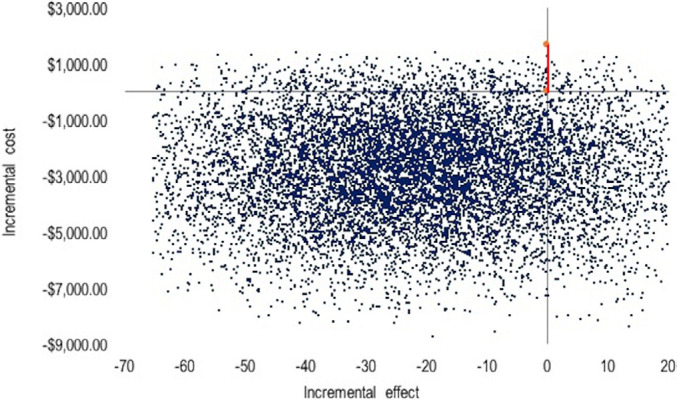
Probabilistic sensitivity analysis for cost-effectiveness per hour of avoided mechanical ventilation with 10,000 interactions.

The probabilistic sensitivity analysis for the cost-effectiveness per hour of mechanical ventilation avoided showed that most of the interactions (80.8%) were in the third quadrant. In this quadrant, the costs and mechanical ventilation duration are lower. Therefore, propofol was, again, the dominant alternative (Figure 3).

**Table 1:** Values used in the sensitivity analysis

Parameter	Base value	Minimum value	Maximum value	Distribution	Reference
Difference in the length of ICU stay (hours) (propofol - midazolam)	-47.97	18.46	-114.40	Normal	Meta-analysis^(7)^
Difference in the mechanical ventilation time (hours) (propofol - midazolam)	-32.68	-22.06	-65.36	Normal	Meta-analysis^(7)^
Cost of ICU stay per day (US$)	1,500.42	597.13	1,848.02	Log-normal	Database of private plans in the state of São Paulo, Brazil^(8)^

ICU - intensive care unit.

## DISCUSSION

In 2018, the Society of Critical Care Medicine (SCCM) published the PADIS guidelines, a revision of the guidelines that were previously published in 2013. In this most current guideline, again, it is recommended that nonbenzodiazepine drugs should be used instead of benzodiazepines for the sedation of patients on mechanical ventilation.

As the recommendation is conditional, it is crucial to determine the impact of the use of nonbenzodiazepine drugs on health costs. To the best of our knowledge, no other cost-effectiveness study has compared the sedation regimen using propofol with the sedation regimen using midazolam.^(9^-^11)^

Our group previously performed a systematic review followed by a meta-analysis comparing the use of propofol (a nonbenzodiazepine) with that of midazolam (a benzodiazepine).^(7)^ Thus, we built a simple decision tree based on the results of our previous study. This study suggests that a propofol-based sedation regimen is cost-effective for sedation in critically ventilated adults in the ICU compared to a midazolam-based sedation regimen.

The clinical differences in the length of stay in the ICU and the duration of mechanical ventilation between the propofol and midazolam regimens incorporated in the model came from the results of a meta-analysis of 23 controlled studies, unlike a previous study whose data came from only two small controlled studies.^(7)^

Our model, similar to other studies that compared sedation regimens with a nonbenzodiazepine with sedation regimens with benzodiazepine, showed that using propofol to sedate critically ill patients under mechanical ventilation is predominately cost saving when compared to midazolam.^(9, 11^-^13)^ These cost savings occur due to the reduced length of ICU stay and the duration of mechanical ventilation. The cost-effectiveness for one hour of ICU stay avoided and for one hour of mechanical ventilation avoided were dominant 95% and 81% of the time, respectively.

The duration of mechanical ventilation is a critical patient-related outcome. In patients hospitalized for more than 24 hours, prolonged use of mechanical ventilation carries a greater risk of complications, especially pneumonia.^(14)^ Ventilator-associated pneumonia (VAP) is a frequent and severe respiratory infection that is often associated with high mortality rates.^(15)^ In a Brazilian private health database, there were 24 cases of VAP for every 1,000 hours under mechanical ventilation.^(16)^ Therefore, based on the present data, we estimated that propofol would reduce 520 cases of VAP in 10,000 patients.

Cost-effectiveness analysis often presents potential limitations.^(17)^ We consulted one database representing only the state of São Paulo and assumed that the data were representative of Brazil’s private health system. The drugs’ cost was included in the daily cost of the ICU stay, and unfortunately, the database consulted did not present the costs for the different drugs separately. Our study did not include the costs associated with possible adverse events related to the drugs under investigation, such as *delirium* or infection. On the other hand, an adverse event related to these drugs would probably increase mechanical ventilation duration, which would appear in the results.^(18)^ We also did not incorporate in our model the daily interruption of sedation or sedation guided by a nursing protocol that could potentially reduce the duration of mechanical ventilation.^(19)^ Finally, as we only evaluated costs related to hospital admission, we could not capture the potential long term effects. This limitation is relevant because prolonged mechanical ventilation and the use of benzodiazepines were associated with the occurrence of *delirium* and postintensive care syndrome, both with a high impact on long-term morbidity, health-related costs, and mortality.^(20, 21)^

## CONCLUSION

From the perspective of the Brazilian private health system, the use of propofol as the first choice sedative for critically ill adult patients treated in the intensive care unit and who need mechanical ventilation for more than 24 hours proved to be cost-saving due to its capacity to reduce the length of intensive care unit stay and the duration of mechanical ventilation. Our results are consistent with the PADIS guidelines of using nonbenzodiazepine drugs for sedation in critically ill, mechanically ventilated adults.
