# Supplementary material for: Uma análise de custo-efetividade de propofol versus midazolam para sedação de pacientes adultos admitidos à unidade de terapia intensiva
Source: Rev Bras Ter Intensiva. 2021 Jul-Sep;33(3):428–33. doi: 10.5935/0103-507X.20210068 (PMC8555397; doi:10.5935/0103-507X.20210068)
Supplement: Supplementary file 1 [file rbti-33-03-0428-suppl01.pdf]

A cost-effectiveness analysis of propofol versus midazolam for the sedation of adult patients admitted to the intensive care unit

Uma análise de custo-efetividade de propofol versus midazolam para sedação de pacientes adultos admitidos à unidade de terapia intensiva

Teresa Raquel Andrade[1], Jorge Ibrain Figueira Salluh[2], Raphaela Garcia[1], Daniela Farah[1], Paulo Sérgio Lucas da Silva[3], Danielle F. Bastos[4], Marcelo Cunio Machado Fonseca[5]

Table 1S - Analgesia management

Author	Propofol drug management	Midazolam drug management	Use of sedation score	Use of analgesia score	Sedation procedures for airway management (e.g., orotracheal aspiration)	Use of mechanical containment	Management of *delirium*
Degauque et al.[(1)]	The mean infusion rate was 1.73mg/kg/hour (1.35 - 1.99)<br>The nursing staff or the intensivist adjusted the infusion rate to the desired sedation level<br>Duration of infusion: 186 hours (50 - 288)	The mean infusion rate was 0.14mg/kg/hour (0.04 - 0.17)<br>The nursing staff or the intensivist adjusted the infusion rate to the desired sedation level<br>Duration of infusion: 155,8 hours (106 - 192)	The authors assessed the level of sedation using the Ramsay scale.<br>Patients requiring ventilation were sedated to Ramsay scale 4 - 6; those needing synchronized intermittent mandatory ventilation and pressure support were sedated to scale 2 - 4, while those just on pressure support were sedated to scale 2 - 3. The overall quality of sedation was assessed by the nursing staff every eight hours using a simplified score: adequate (1), inappropriate (2), or patient resisting ventilation (3)	Not available	Not available	Not available	Not available
Kress et al.[(2)]	Propofol continuous infusion at 5µg/kg/minute; may increase infusion in 5 - 10µg/kg/minute increments every 2 minutes until adequate sedation achieved<br>Morphine sulfate 2 - 10mg IVP prn<br>Morphine sulfate continuous infusion at 1 - 5mg/hour	Midazolam 0.5 - 5mg IVP every 1 - 5 minutes prn. Midazolam continuous infusion at 1 - 2mg/hour; may increase continuous infusion in 1 - 2mg/hour increments until adequate sedation achieved<br>Morphine sulfate 2 - 10mg IVP prn<br>Morphine sulfate continuous infusion at 1 - 5mg/hour	Nurses titrated infusions according to the Ramsay sedation score of 3 or 4	prn dosing was based on nurse's assessment of pain: 1 = extreme pain, 2 = severe pain, 3 = moderate pain, 4 = slight pain, 5 = no pain<br>Morphine was administered initially in response to a pain score of 1 to 4 until the pain was viewed as adequately controlled	Not available	Not available	Not available

continue...

...continuation

Author	Propofol drug management	Midazolam drug management	Use of sedation score	Use of analgesia score	Sedation procedures for airway management (e.g., orotracheal aspiration)	Use of mechanical containment	Management of *delirium*
Mesnil et al.[3]	IV propofol 2% was initiated at 2mg/kg/hour. Further adjustments were made by the patient's nurse for Ramsay scale values of 3 - 4 with an assessment of the score every 10 min and adaptation in steps of 0.1mg kg-1 h-1 for propofol if necessary<br>Duration of sedation: 57 hours (35 - 89)	IV midazolam was initiated at 0,1mg/kg/hour. Further adjustments were made by the patient's nurse for Ramsay scale values of 3 - 4 with an assessment of the score every 10 min and adaptation in steps of 0.05mg kg-1 h-1 for midazolam if necessary<br>Duration of sedation: 50 hours (38 - 71)	Sedation was evaluated every 10 minutes by the nurse in charge of the patient until Ramsay's score of 3 - 4 was reached, then every hour	Analgesia was achieved in the three groups by continuous infusion of remifentanil, started at 0.15µg kg-1 min-1 intravenously. Further adjustments of remifentanil infusion were made for pain scale values of 1. Analgesia levels were evaluated every hour	Not available	Not available	Not available
Ruokonen et al.[4]	Propofol was infused at 2.4mg/kg/hour for 1 hour and then adjusted stepwise at 0.8, 1.6, 2.4, 3.2, and 4.0mg/kg/hour<br>The initial dose could be reduced if considered necessary by the treating clinician<br>Duration of drug administration: 61 hours (15 - 256)	Depending on standard care at the time of randomization, midazolam was given either as intravenous boluses (1 - 2mg), starting at three boluses per hour for one hour, and after that 1 - 4 boluses per hour, and if not sufficient as a continuous infusion of 0.2mg/kg/hour, or as a continuous infusion at 0.12mg/kg/hour for 1 hour, followed by adjustments at 0.04, 0.08, 0.12, 0.16, and 0.20mg/kg/hour. The initial dose could be reduced if considered necessary by the treating clinician<br>Duration of drug administration: 27 hours (4 - 105)	Drugs titrated to target sedation range using RASS	Not available	Not available	Not available	Not available

continue...

...continuation

Author	Propofol drug management	Midazolam drug management	Use of sedation score	Use of analgesia score	Sedation procedures for airway management (e.g., orotracheal aspiration)	Use of mechanical containment	Management of *delirium*
Sanchez-Izquierdo-Riera et al.[5]	Continuous IV administration of propofol at the rate of 1.5mg/kg/hour. The dose rate could be increased according to the patient's sedation level up to a maximal dose of 6mg/kg/hour<br>The requirement for larger doses was considered a therapeutic failure. In this case, the patient was withdrawn from the protocol, and other sedatives' administration was allowed<br>Ill patients received morphine chloride as an analgesic at a dose of 0.02 - 0.04mg/kg/hour	Continuous IV administration of midazolam at the rate of 0.1mg/kg/hour. The dose rate was increased according to the patient's sedation level, up to a maximal dose of 0.35mg/kg/hour<br>The requirement of doses larger than maximal was considered a therapeutic failure. In this case, the patient was withdrawn from the protocol, and other sedatives' administration was allowed<br>The other midazolam group received continuous IV administration of midazolam started at 0.1mg/kg/hour<br>The dose rate was increased according to the patient's sedation level up to a maximal dose of 0.2mg/kg/hour. If a larger dose was required, propofol was infused at a variable dose rate (1.5 - 3mg/kg/hour)<br>All patients received morphine chloride as an analgesic at a dose of 0.02 - 0.04mg/kg/hour	The authors assessed the level of sedation employing a simplified Ramsay scale. The different drug dose rates were modified to achieve a sedation level of 3 or 4. The level of sedation was determined by the nursing staff at least three times a day	Not available	Not available	Not available	Not available

continue...

...continuation

Author	Propofol drug management	Midazolam drug management	Use of sedation score	Use of analgesia score	Sedation procedures for airway management (e.g., orotracheal aspiration)	Use of mechanical containment	Management of *delirium*
Sandiumenge Camps et al.[6]	The 2% propofol group received a continuous intravenous administration of 1.5mg/kg/hour 2% propofol. Increasing infusion rates were administered according to the patient sedation level up to 6mg/kg/hour (typically recommended infusion rates). Higher requirements than the maximal dosage were considered a therapeutic failure. In this case, the patient was withdrawn from the trial, and other sedatives were allowed<br>Duration of sedation: 5.2 days ± 3.1	The midazolam group received a continuous intravenous administration of 0.1mg/kg/hour midazolam. The infusion rate was increased according to the patient's sedation level to a maximum of 0.35mg/kg/hour (typically recommended infusion rates)<br>The need for higher requirements of this sedative was considered a therapeutic failure; the patient was withdrawn from the study, and the administration of other sedatives was allowed<br>Duration of sedation: 6.6 days ± 4.6	Drug dosages were adjusted to achieve a 3 - 4 level of sedation (5 -6 on the Ramsay scale). This sedation level was determined hourly by trained nursing staff. In patients requiring muscle relaxation because of intracranial hypertension or severe respiratory failure, the sedation level was determined 3 hours after the last administration of muscle relaxant. If discontinuation of relaxation was not possible, a simple scale based on clinical signs, the Evans scale, was used. This scale numerically grades from 0 to 3 points each of a set of variables, such as a rise in blood pressure over the base level, a rise in heart rate over the base rate, the presence or absence of perspiration, and the presence or absence of tearing. Any total score of 3 points was considered inadequate sedation. Any patient needing barbiturate coma induction to control intracranial pressure was removed from the protocol as soon as this action was taken	Not available	Not available	Not available	Not available
Weinbroum et al.[7]	A loading dose of propofol 1.3 ± 0.2mg/kg/hour was administered, followed by continuous infusion, titrated to achieve a predetermined sedation score. Sedation was continued as long as clinically indicated<br>Duration of sedation: 99 hours ± 15	A loading dose of midazolam 0.11 ± 0.02 (SEM) mg/kg/h was administered, followed by continuous infusion, titrated to achieve a predetermined sedation score. Sedation was continued as long as clinically indicated<br>Duration of sedation: 141 hours ± 27	The dose was titrated to achieve a level of sedation of 2 - 3 on a five-point sedation scale: 1 = awake; 2 = drowsy, responds to verbal stimulation; 3 = asleep but awakens to mild tactile stimulation; 4 = asleep, responds to painful stimulation only; 5 = deeply asleep, does not respond to painful stimulation<br>Patients were also evaluated at each sedation level as to whether they were agitated (-) or calm (+)	Not available	Not available	Not available	Not available

continue...

...continuation

Author	Propofol drug management	Midazolam drug management	Use of sedation score	Use of analgesia score	Sedation procedures for airway management (e.g., orotracheal aspiration)	Use of mechanical containment	Management of *delirium*
Zhou et al.[8]	Propofol group: received an infusion bolus of 0.50 - 3.00mg/kg and continuous infusion of 0.50 - 3.00mg/kg/hour, with the dosage adjusted to achieve the desired level of sedation. All patients received continuous intravenous fentanyl for analgesia with a bolus dosage of 1 - 2µg/kg and a maintenance dosage of 1 - 2µg/kg/hour<br>Duration of sedation: 120 hours (interquartile range 70)	Midazolam group: were treated within infusion bolus of 0.03 - 0.30mg/kg and continuous infusion of 0.04 to 0.20mg/kg/hour, with the dosage adjusted to achieve the desired sedation level. All patients received continuous intravenous fentanyl for analgesia with a bolus dosage of 1 - 2µg/kg and a maintenance dosage of 1 - 2 µg/kg/hour<br>Duration of sedation: 142 hours (interquartile range 94.5)	The Riker sedation–agitation scale (SAS) was used to assess each group's sedation quality. According to our local sedation procedure, the nurses continuously monitored the sedation depth and adjusted the dosages of sedative and analgesic drugs to maintain the sedation target level to 3 or 4 points of degree on the SAS scale. SAS scores were recorded every 4 hours (or more frequently when indicated) by the nursing staff to ensure correct titration of the sedative infusion	No available	Not available	Not available	*Delirium* was not assessed as it was left to the treating physicians

IVP - intravenous push; prn - *pro re nata*; RASS - Richmond Agitation-Sedation Scale; SEM - standard error of the mean.

REFERENCES

1. Degauque C, Dupuis A. A study to compare the use of propofol and midazolam for the sedation of patients with acute respiratory failure. J Drug Dev. 1991;4(Suppl 3):95-7.
2. Kress JP, Pohlman AS, Hall JB. Effects of sedative interruption in critically ill, mechanically ventilated patients receiving midazolam or propofol. J Clin Outcomes Manag. 2001;8(2):33-9.
3. Mesnil M, Capdevila X, Bringuier S, Trine PO, Falquet Y, Charbit J, et al. Long-term sedation in intensive care unit: a randomized comparison between inhaled sevoflurane and intravenous propofol or midazolam. Intensive Care Med. 2011;37(6):933-41.
4. Ruokonen E, Parviainen I, Jakob SM, Nunes S, Kaukonen M, Shepherd ST, Sarapohja T, Bratty JR, Takala J; "Dexmedetomidine for Continuous Sedation" Investigators. Dexmedetomidine versus propofol/midazolam for long-term sedation during mechanical ventilation. Intensive Care Med. 2009;35(2):282-90.
5. Sanchez-Izquierdo-Riera JA, Caballero-Cubedo RE, Perez-Vela JL, Ambros-Checa A, Cantalapiedra-Santiago JA, Alted-Lopez E. Propofol versus midazolam: safety and efficacy for sedating the severe trauma patient. Anesth Analg. 1998;86(6):1219-24.
6. Sandiumenge-Camps A, Sanchez-Izquierdo Riera JA, Toral Vazquez D, Sa Borges M, Peinado Rodriguez P, Alted Lopez E. Midazolam and 2% propofol in long-term sedation of traumatized, critically ill patients: efficacy and safety comparison: Crit Care Med. 2000;28(11):3612-9.
7. Weinbroum AA, Halptern P, Rudick V, Sorkine P, Freedman M, Geller E. Midazolam versus propofol for long-term sedation in the ICU: a randomized prospective comparison. Intensive Care Med. 1997;23(12):1258-63.
8. Zhou Y, Jin X, Kang Y, Liang G, Liu T, Deng N. Midazolam and propofol used alone or sequentially for long-term sedation in critically ill, mechanically ventilated patients: a prospective, randomized study. Crit Care. 2014;18(3):R122.
